# Supplementary material for: Evaluating the Effectiveness of Calcium Silicate in Enhancing Soybean Growth and Yield
Source: Plants (Basel). 2023 May 31;12(11):2190. doi: 10.3390/plants12112190 (PMC10255688; doi:10.3390/plants12112190)
Supplement: Supplementary file 1 [file plants-12-02190-s001.zip › plants-2359423-supplementary.pdf]

Table S1. Information of chemical composition of Soil at two geographic locations.

Parameters	Gunwi	Gyeongsan
pH (water)	6.0	6.2
*Cation Exchange Capacity (mmol(c)/kg)	158	190
Total Nitrogen (cg/kg)	299	279
Organic Carbon Density (g/dm^3)	573	566
Bulky density (cg/cm^3)	128	131
Clay (%)	26.8	28.6
Sand (%)	37.0	30.6
Silt (%)	36.3	40.8

Sampling depth (5-15 cm)

Table S2. Year-wise precipitation and temperature data in the growing season at two geographic locations.

	TP (mm)/ ASAT(°C) 2022		TP (mm)/ ASAT(°C) 2021		TP (mm)/ASAT (°C) 2020	
	**Gyeongsan**	**Gunwi**	**Gyeongsan**	**Gunwi**	**Gyeongsan**	**Gunwi**
**May**	8/16.85	5/16.85	139/15.85	180/14.85	43/16.85	55/16.85
**June**	125/21.85	140/20.85	98/20.85	92/20.85	152/21.85	182/20.85
**July**	168/24.85	168/24.85	233/24.85	195/23.85	344/21.85	290/20.85
**August**	184/24.85	299/23.85	264/24.85	311/22.85	287/25.85	327/25.85
**September**	161/19.85	124/18.85	80/23.85	86/19.85	260/18.85	171/18.85
**October**	61/13.85	69/12.85	44/14.85	65/13.85	55/13.85	46/12.85

TP; Total precipitation in (mm), ASAT; Average surface air temperature in (°C).

Reference

Beaudoing, H.; Rodell, M. NASA/GSFC/HSL (2020), GLDAS Noah Land Surface Model L4 monthly 0.25 x 0.25 degree V2.1, Greenbelt, Maryland, USA, Goddard Earth Sciences Data and Information Services Center (GES DISC). Available online: https://disc.gsfc.nasa.gov/datasets/GLDAS_NOAH025_M_2.1/summary (accessed on March 1 2023).
